# MCL-1 dependency as a novel vulnerability for aggressive B cell lymphomas

**DOI:** 10.1038/s41408-020-00402-2

**Published:** 2021-01-14

**Authors:** Michelle Y. Wang, Tao Li, Yuan Ren, Bijal D. Shah, Tint Lwin, Jing Gao, Kenneth H. Shain, Wei Zhang, Xiaohong Zhao, Jianguo Tao

**Affiliations:** 1 Chemical Biology and Molecular Medicine Program, H. Lee Moffitt Cancer Center & Research Institute, Tampa, FL 33612 USA; 2 Department of Malignant Hematology, H. Lee Moffitt Cancer Center & Research Institute, Tampa, FL 33612 USA; 3 Department of Tumor Biology, H. Lee Moffitt Cancer Center & Research Institute, Tampa, FL 33612 USA; 4 Department of Computer Science, University of Central Florida, Orlando, FL 32816 USA; 5 Department of Hematopathology and Laboratory Medicine, H. Lee Moffitt Cancer Center & Research Institute, Tampa, FL 33612 USA

**Keywords:** Cancer genomics, Pharmacogenomics, B-cell lymphoma, Cancer therapeutic resistance

Dear Editor,

Mantle cell lymphoma (MCL) and diffuse large B cell lymphoma (DLBCL) are aggressive hematologic malignancies characterized by the accumulation of lymphoid cells defective in cell apoptosis biology and function^1^. The anti-apoptotic B cell lymphoma 2 (BCL-2) family proteins are pivotal regulators of the mitochondrial apoptotic pathway and genetic aberrations in these genes are associated with lymphomagenesis and chemotherapeutic resistance. These anti-apoptotic proteins, most notably BCL-2 and myeloid cell leukemia 1 (MCL-1), promote the survival of lymphoma cells by counteracting the activity of pro-apoptotic proteins such as BCL-2-like protein 11 (BCL2L11, also known as BIM)^2–4^. Pharmacological targeting of BCL-2 is highly effective in certain B cell lymphomas, but de novo and acquired resistance to BCL-2 inhibitor monotherapy often develop, especially in aggressive B cell lymphomas. Moreover, the other major anti-apoptotic BCL-2 family proteins, BCL-XL and MCL-1, are demonstrated to be the main determinants of resistance to venetoclax^5^. Additionally, MCL-1 is recurrently highly expressed in various kinds of non-Hodgkin’s B cell lymphomas^6^. Collectively, these data support the hypothesis that MCL-1 plays a central role in B cell lymphoma progression and drug resistance. Pharmacologically targeting MCL-1, therefore, represents an attractive strategy to combat these lymphomas. To this end, there is a great need to develop and apply selective small-molecule MCL-1 inhibitors as part of a first-line lymphoma therapy or upon emergence of tumor resistance characterized by upregulation of MCL-1.

Here, we exploited the MCL-1 dependency in MCL and DLBCL by implementing pharmacogenomic and chemical proteomic approaches^5,7^ to investigate the molecular drug response and resistance mechanism to MCL-1 inhibitors. We demonstrated that transcriptome and kinome reprograming linked to the MEK and ERK pathways contribute to MCL-1 inhibitor resistance via regulation of the BCL-2 family profile. Further analysis revealed synergistic activity of MCL-1 inhibitors in combinations with inhibitors of MEK, ERK, and BCR in MCL-1 inhibitor-resistant MCL/DLBCL lines and primary samples. These results provide a strong rationale for further evaluation of MCL-1 inhibitor in combination with established therapy in the clinical setting and highlight a potential strategy for overcoming MCL-1 inhibitor resistance.

First, we affirmed the dependency of MCL-1 in MCL and DLBCL survival and growth. We performed cell-viability assays in a panel of MCL and DLBCL lines for their susceptibility to specific MCL-1 inhibitor, S63845. As shown in Fig. 1A, most MCL and DLBCL lines were highly sensitive to S63845. Additionally, MCL-1 inhibition with S63845 triggered dramatic PARP cleavage in MCL/DLBCL lines and primary samples, indicating that the compound’s inhibitory effect was attributed to induced mitochondrial-mediated apoptosis (Fig. S1A). These results are in line with the notion that MCL and DLBCL typically have high expression of MCL-1 as well as BCL-2 (refs. ^6,8^). While targeting MCL-1 appears to be a viable therapeutic strategy^9^, previous clinical and pre-clinical data suggest that treatment with single-agent anti-BCL-2 family member therapy is associated with the rapid acquisition of resistance.

**Fig. 1:**
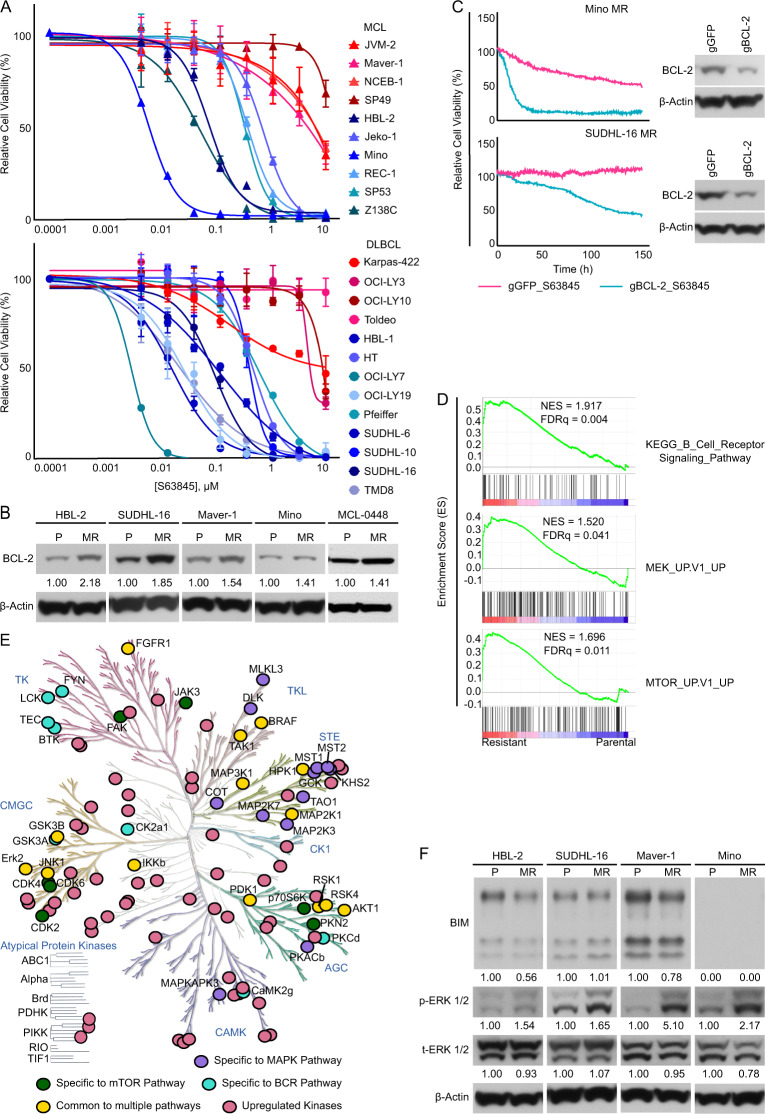
MCL-1 dependency and development of resistance to MCL-1 inhibition in mantle cell lymphoma (MCL) and diffuse large B cell lymphoma (DLBCL). **A** Dose–response curves of MCL (top) and DLBCL (bottom) cell lines treated with S63845 for 72 h. Data are shown as mean ± SD, *n* = 3 technical replicates for each cell line. **B** Western blot analysis of BCL-2 protein expression in paired parental (P) and S63845-resistant (MR) cell lines. Band density was quantified and each MR cell line was normalized to its parental cell line. **C** Image-based cell-viability assays of MR cells with and without BCL-2 knockdown in response to S63845 (top: 10 μM, bottom: 1.11 μM). Inset: western blot confirming BCL-2 knockdown in each MR cell line. **D** Gene set enrichment analysis (GSEA) enrichment score plots of selected pathways from KEGG (C2) and Oncogenic Signatures (C6) gene sets from MSigDB. Genes were ranked according to their gene expression fold change between SUDHL-16 MR and parental cells. NES normalized enrichment score, FDRq false discovery rate adjusted *p* value. **E** Kinome tree representation of kinases with activity significantly upregulated in SUDHL-16 MR cells compared to parental cells. Upregulated kinases defined as having fold change of 1.5. Labeled kinases belong to the BCR, MEK–MAPK, and PI3K–AKT/mTOR pathways. **F** Western blot analysis of paired parental, and MR cells. p-ERK phosphorylated ERK, t-ERK total ERK. Band density was quantified for each protein and each MR cell line was normalized to its parental cell line. Data shown in **A**, **B**, and **F** are representative of at least three independent experiments.

In anticipation of the evolution of MCL-1 inhibitor resistance, we developed MCL-1 resistance (MR) models by treating S63845-sensitive cell lines with high doses of S63845 for an extended period in MCL, DLBCL, and MCL-derived lines (10 × IC_50_ for 3 months). Though parental lines were exquisitely sensitive to S63845, the IC_50_s for S63845 were more than 10–100-fold higher in all derived resistant lines (Fig. S1B). Similarly, these MR lines showed a comparable level of resistance to the chemically distinct MCL-1 inhibitor, AZD5991 (Fig. S1C). Using these models, we next investigated the cellular pathways responsible for the MCL-1 inhibitor-resistant state. We first examined the expression of other anti-apoptotic BCL-2 family members. Western blot analysis demonstrated that MR lymphoma cells exhibited significantly increased expression of BCL-2, but only minimal changes in BCL-XL compared to parental cells in these MCL and DLBCL lines and primary sample (Figs. 1B and S1D). To determine the functional role of BCL-2 in conferring S63845 resistance, we applied CRISPR/Cas9 editing to knockdown (KD) BCL-2 expression in resistant cells (SUDHL-16, Mino). These experiments revealed that BCL-2-KD MR cells exhibited loss of cell viability (Fig. S1E). Importantly, BCL-2-KD reversed the resistance to MCL-1 inhibitor in S63845-selected cell lines. Consistent with a reduction in BCL-2 dependence, BCL-2-KD MR lines also demonstrated a decreased sensitivity to BCL-2 antagonist, ABT-199 (Figs. 1C and S1F). However, the decrease in ABT-199 sensitivity was not as dramatic as the increase in S63845 sensitivity, which we attributed to the remaining expression of BCL-2 as well as the strong potency and selectivity of ABT-199. To further confirm the importance of BCL-2 in MCL-1 inhibitor resistance, we tested the effect of ABT-199 in MR lines and found that similar to BCL-2 KD, BCL-2 inhibition enhanced the potency of S63845 in these cells (Fig. S1G). Together, these data indicated that altered dependency on BCL-2 contributed to S63845 resistance.

Next, we performed RNA-sequencing on parental and resistant SUDHL-16 cells. Analysis of the transcriptomes of the sensitive and resistant cells revealed a set of differentially expressed genes in the resistant phenotype, with 359 upregulated genes and 194 downregulated genes in MR (Fig. S1H). Implementation of gene set enrichment analysis (GSEA) revealed a significant positive enrichment of MEK–MAPK, mTOR, and BCR pathways in SUDHL-16 MR cells (Fig. 1D). Chemical activity-based proteomic profiling^7,10^ was performed in parallel on paired SUDHL-16-sensitive and -resistant cells. Consistent with our previous findings^5,10^, the gene expression programs identified by RNA-seq translated to kinome reprogramming in SUDHL-16 MR cells with the activation of many kinase pathways, including the BTK, MEK, and ERK pathways (Fig. 1E). Increased MEK/ERK signaling was confirmed by western blot, illustrating increased ERK phosphorylation and BIM downregulation in S63845-resistant lines (SUDHL-16, HBL-2, Mino, Marver-1, Fig. 1F). Collectively, these data demonstrated that transcriptome and kinome reprogramming in MR lymphoma cells were associated with upregulation of the MEK–ERK, AKT–mTOR, and BCR pathways, which may converge on BCL-2 and BIM to promote S63845 resistance.

To determine the functional role of transcriptome and kinome reprogramming in BCL-2 family protein expression and S63845 resistance, a drug sensitivity screen composed of 31 small-molecule kinase inhibitors^5,7,10^ was performed on paired parental and MR cells (SUDHL-16, Mino, HBL-2). Additionally, we used S63845 as an “anchor” to determine the key signaling inhibitors that reinstated S63845 sensitivity in resistant cells. In line with our transcriptome and kinome results, MR cells exhibited higher sensitivity to inhibitors of the MEK (Trametinib), ERK (SCH772984), and BCR pathways (Ibrutinib, R406, Fig. S2A). Importantly, the combination of S63845 with these inhibitors showed enhanced potencies against MR lines (Fig. 2A). Cell viability and colony formation assays validated synergistic effects of the combination of S63845 with Trametinib or SCH772984 in all tested MR lines (Fig. S2B, C). Similar enhanced effects of these combination treatments were observed in MCL and DLBCL cell lines with pre-existing MCL-1 inhibitor resistance (Figs. 1A and S2D), indicating that this is an effective approach to combat both de novo and acquired resistance to MCL-1 inhibition.

**Fig. 2:**
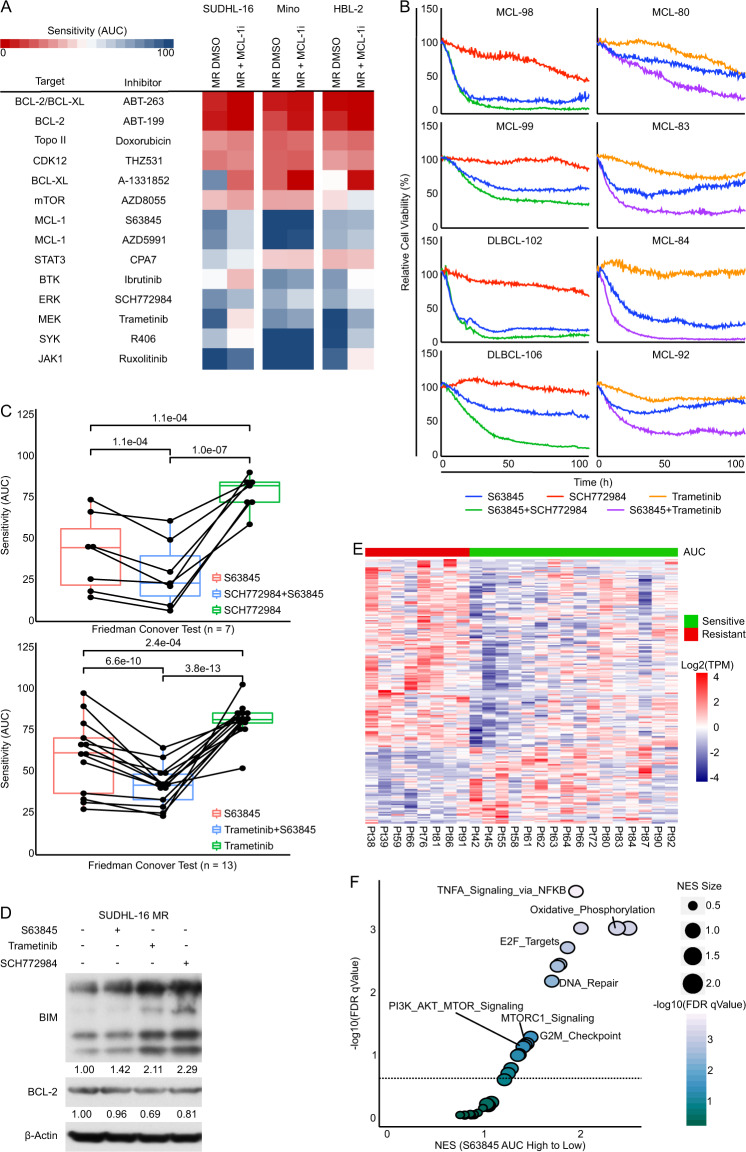
MCL-1 as a novel vulnerability for combination therapy in mantle cell lymphoma (MCL) and diffuse large B cell lymphoma (DLBCL). **A** Heatmap of AUCs of drug–response curves from image-based cell-viability assays performed in MR cells with and without the presence of S63845 as an anchor drug for indicated cell lines. **B** Left: Image-based cell-viability assays of primary MCL and DLBCL samples co-cultured with stroma cells in response to treatment with S63845, SCH772984, or S63845+SCH772984. Right: Image-based cell-viability assays of primary patient samples co-cultured with stroma cells in response to treatment with S63845, Trametinib, or S63845+Trametinib. **C** Quartile box plots of drug sensitivity as determined by AUCs of image-based cell-viability assays in primary MCL samples. *P* values between treatment groups were calculated with the Friedman Conover Test. Top: Primary samples treated with S63845, SCH772984, or S63845+SCH772984. *n* = 7. Bottom: Primary samples treated with S63845, Trametinib, or S63845+Trametinib. *n* = 13. **D** Western blot analysis of BCL-2 family proteins in MR cells after 48 h treatment with S63845 (2 μM), Trametinib (2 μM), or SCH772984 (2 μM). **E** Heatmap of top differentially expressed genes between primary S63845-resistant and S63845-sensitive patient samples defined by AUCs calculated from image-based cell-viability assays. **F** Scatterplot of GSEA-positive NES of the Hallmarks gene set from MsigDB in primary patient samples phenotypically characterized by S63845 sensitivity as determined by AUCs calculated from image-based cell-viability assays. FDRq cut-off of 0.25. Data shown in **A** and **D** are representative of at least three independent experiments.

Next, we capitalized on a novel ex vivo microfluidic cell-based platform^5,7,10^ to assess the response of a panel of kinase inhibitors as well as the combination of S63845 with these kinase inhibitors in a 3D reconstructed tumor microenvironment on primary MCL samples collected from lymph node and peripheral blood^11,12^. Again, we observed enhanced effects of S63845 combined with Trametinib or SCH772984 and synergistic effects of both combinations in S63845-resistant primary MCL samples (Fig. 2B, C). Mechanistically, western blot analysis showed that both MEK and ERK inhibition induced BCL-2 downregulation and BIM upregulation in MR lines (SUDHL-16). Furthermore, the combination of S63845 with Trametinib or SCH772984 triggered increased PARP cleavage compared to S63845, MEK, or ERK inhibitor alone in SUDHL-16 and Mino-resistant models (Figs. 2D and S2E). These data indicate that kinome reprograming linked to the MEK and ERK pathways contributed to MCL-1 inhibitor resistance via regulation of the BCL-2 family profile (BCL-2, BIM), and represent a novel vulnerability in MCL-1 inhibitor-resistant lymphoma.

Finally, we integrated RNA-seq data with drug response profiling data for 24 primary MCL samples. We determined drug sensitivity by calculating the area under curve (AUC) and the half maximal effective concentration (EC_50_) of each drug for every patient. As shown in Fig. 2E, using the AUC of S63845, we segregated these primary samples into responders (*n* = 10) and non-responders (*n* = 14) and correlated S63845 response with the gene expression profile of each sample. Consistent with our in vitro results, the investigation of differentially expressed genes of these two response groups by GSEA revealed enrichment of mTOR, MEK–ERK, and BCR pathway-regulated genes in S63845-resistant primary samples (Figs. 2F and S2F). Collectively, these data identify a targetable MEK–ERK dependent regulation of MCL-1 inhibitor resistance with significant translational potential in future clinical combinations trials.

In conclusion, we identified MCL-1 as a key determinant of cell survival and growth in MCL and DLBCL lymphomas, which conferred exquisite sensitivity to MCL-1 inhibition. Using pharmacogenomics and chemical proteomics, we identified that MEK–ERK and BCR pathway activation drive MCL-1 inhibitor resistance in these lymphomas via transcriptional upregulation of BCL-2 (refs. ^13,14^) and post-translational downregulation of BIM^15^. To this end, combinatorial therapies of MCL-1 inhibitor with MEK, ERK, or BCR inhibitors (to decrease BCL-2 and increase BIM) exhibited potent synergistic effects against MCL-1 inhibitor-resistant lymphoma ex vivo and in vivo. Together, these studies provide a rationale for MCL-1 inhibition in MCL/DLBCL and present MCL-1 inhibition-based combination strategies to MCL-1-dependent and MCL-1 inhibitor-resistant B cell lymphomas.

## Supplementary information

Supplementary Information

## References

[1] Campo E (2017). Pathology and classification of aggressive mature B-cell lymphomas. Hematol. Oncol..

[2] Sharon D (2019). Inhibition of mitochondrial translation overcomes venetoclax resistance in AML through activation of the integrated stress response. Sci. Transl. Med..

[3] Klanova ,M, Klener ,P (2020). BCL-2 Proteins in Pathogenesis and Therapy of B-Cell Non-Hodgkin Lymphomas. Cancers (Basel).

[4] Merino D (2018). BH3-mimetic drugs: blazing the trail for new cancer medicines. Cancer Cell.

[5] Zhao X (2019). BCL2 amplicon loss and transcriptional remodeling drives ABT-199 resistance in B cell lymphoma models. Cancer Cell.

[6] de Jong MRW (2019). Heterogeneous Pattern of Dependence on Anti-Apoptotic BCL-2 Family Proteins upon CHOP Treatment in Diffuse Large B-Cell Lymphoma. Int J Mol Sci.

[7] Ren Y (2018). PLK1 stabilizes a MYC-dependent kinase network in aggressive B cell lymphomas. J. Clin. Invest..

[8] Jiang H (2019). Venetoclax as a single agent and in combination with PI3K-MTOR1/2 kinase inhibitors against ibrutinib sensitive and resistant mantle cell lymphoma. Br. J. Haematol..

[9] Dengler MA (2020). Potent efficacy of MCL-1 inhibitor-based therapies in preclinical models of mantle cell lymphoma. Oncogene.

[10] Zhao X (2017). Unification of de novo and acquired ibrutinib resistance in mantle cell lymphoma. Nat. Commun..

[11] Khin ZP (2014). A preclinical assay for chemosensitivity in multiple myeloma. Cancer Res..

[12] Silva, A., Jacobson, T., Meads, M., Distler, A. & Shain, K. An organotypic high throughput system for characterization of drug sensitivity of primary multiple myeloma cells. *J. Vis. Exp.* (101), e53070 (2015). 10.3791/53070 PMC4544963 26274375

[13] Subramanian M, Shaha C (2007). Up-regulation of Bcl-2 through ERK phosphorylation is associated with human macrophage survival in an estrogen microenvironment. J. Immunol.

[14] Wang C (2015). Up-regulation of Bcl-2 by CD147 through ERK activation results in abnormal cell survival in human endometriosis. J. Clin. Endocrinol. Metab..

[15] Craxton A, Draves KE, Gruppi A, Clark EA (2005). BAFF regulates B cell survival by downregulating the BH3-only family member Bim via the ERK pathway. J. Exp. Med..

