# Supplementary material for: MCL-1 dependency as a novel vulnerability for aggressive B cell lymphomas
Source: Blood Cancer J. 2021 Jan 14;11(1):14. doi: 10.1038/s41408-020-00402-2 (PMC7809345; doi:10.1038/s41408-020-00402-2)
Supplement: Supplementary file 1 — Supplementary Information [file 41408_2020_402_MOESM1_ESM.pdf]

Supplementary Information for Manuscript "MCL-1 dependency as a novel vulnerability for aggressive B-cell lymphomas"

Summary:

This supplement contains the Materials and Methods section as well as two supplementary figures with accompanying figure legends related to the two main figures submitted with this manuscript.

This PDF file contains:

Materials and Methods

Supplementary Figures and Figure Legends

Materials and Methods

Materials

Patients and Tumor Specimens

The primary samples from MCL patients were obtained from fresh biopsy-derived lymphoma tissues (lymph nodes) and from peripheral blood following informed consent from patients and approval by the Moffitt Cancer Center/University of South Florida Institutional Review Board. For preparation of viable, sterile, single cell suspensions, the lymph node tissue was diced and forced through a cell strainer into RPMI-1640 tissue culture medium. Cells, obtained after low-speed centrifugation, were re-suspended in media. Lymphoma cells from peripheral blood were isolated by Ficoll-Plaque purification, and only lymphoma samples that had greater than 80% tumor cells were used for experiments.

Cell Lines

Mantle cell lymphoma (MCL) and diffuse large B-cell lymphoma (DLBCL) cells lines SUDHL-16, Mino, Maver-1, and HBL-2 were purchased from ATCC. MCL patient derived cell line MCL-0448 was developed in house by culturing a patient specimen in RPMI-1640 (Gibco-Invitrogen) with penicillin (100 U/ml) and streptomycin (100 µg/ml) and maintained at 37°C in 5% CO_2 until cells started to proliferate. These cells and their S63845-resistant derivatives were cultured in RPMI-1640 with penicillin and streptomycin and maintained at 37°C in 5% CO_2. Cell lines were routinely tested for mycoplasma using the Universal Mycoplasma Detection Kit from ATCC.

Study Approval

The human specimen studies presented were approved by the Moffitt/University of South Florida Institutional Review Board and patients provided signed informed consent forms.

Antibodies

For western blotting, the following antibodies were used: c-PARP (Cell Signaling Technologies, 5625), BCL-2 (Cell Signaling Technologies, 2872), BIM (Cell Signaling Technologies, 2933), BCL-XL (Cell Signaling Technologies, 2762), p-ERK1/2 (Cell Signaling Technologies, 9101), ERK1/2 (Cell Signaling Technologies, 9102), MCL-1 (Santa Cruz Biotechnology, sc-819) and Actin (Santa Cruz Biotechnology, sc-47778HRP).

Small Molecule Inhibitors

The following kinase inhibitors were used in drug screening and cell-based drug screening assays: A-1331852 (Selleckchem, S7801), ABT-199 (Selleckchem, S8048), ABT-263 (Selleckchem, S1001), AZD4573 (Selleckchem, S8719), AZD5991 (Selleckchem, S8643), AZD7762 (Selleckchem, S1532), AZD8055 (Selleckchem, S1555), Bendamustine (TargetMol, T0095), Bortezomib (Selleckchem, S1013), Carfilzomib (Selleckchem, S2853), CPA7 (DC Chemicals, DC12009), Dinaciclib (Selleckchem, S2768), Doxorubicin (Selleckchem, S1208), Ibrutinib (Selleckchem, S2680), INCB054329 (Incyte Corporation, Wilmington, DE), INCB052793 (Incyte Corporation, Wilmington, DE), Lumpib (Selleckchem, S1069), NVP-2 (MedChemExpress, HY-12214A), Olaparib (Selleckchem, S1060), R406 (Selleckchem, S2194), Ruxolitinib (Selleckchem, S1378), S63845 (ApexBio, A8737), SCH772984 (Selleckchem, S7101), SR-4835 (Moffitt Cancer Center Derek Duckett Lab), THZ1

(Dana-Farber Cancer Institute Nathanael Gray Lab (1)), THZ531 (Dana Farber Cancer Institute Nathanael Gray Lab), Trametinib (Selleckchem, S2673), VE821 (Selleckchem, S8007), Volasertib (Selleckchem, S2235).

Methods

Generation of S63845 Resistant Cell Lines

To establish the S63845 resistant cell lines, MCL and DLBCL cells (HBL-2, Mino, Maver-1, SUDHL-16, and MCL-0448) were grown in RPMI-1640 medium with 10% FBS and treated with vehicle control or escalating doses of S63845 for approximately 3 months. Before exposing cells to S63845, cells were maintained in suspension. Trypan blue staining and CCK8 viability assay in suspension cells were used to determine viability every 2-3 weeks, and S63845 concentrations were increased if the viability of cells was >65%. If cell viability was <65%, populations were expanded in suspension in drug-free media and then re-exposed to the same concentration of S63845. After ~90 days, drug-resistant variants emerged, and IC_{50}s were determined every two weeks. Following another 30 days of selection, stable variants emerged, referred to as MR. Drug-resistant variants were maintained in drug free media for 3 days before being used in experiments. Cells exposed to DMSO were maintained in parallel and used for comparison with their respective drug-resistant cell lines. We used at least 10 X IC_{50} (ranging from 10 to 100 fold) as the threshold to define resistance.

Colony Formation Assays

For the colony formation assay, 2×10^3 SUDHL-16 parental and MR cells (0.05ml) were added to 0.5 ml MethoCult® (STEMCELL, 4034) per well in 24-well plates. Cells were treated with DMSO, S63845, Trametinib, SCH772984, S63845+Trametinib, or

S63845+SCH772984 with each condition having triplicate wells. After 7, 14, 21, 28, and 35 days in culture, the number of colonies were counted and pictures of the colonies were taken.

CRISPR/Cas9 Editing of BCL-2

For CRISPR/Cas9 editing of BCL2, gRNAs targeting BCL-2 or GFP were cloned into a vector encoding espCas9. Briefly, lentiviral particles were generated by transfection of HEK-293T cells with pLentiCRISPR V2 constructs and the packaging vectors pVSVg (AddGene, 8454) and psPAX2 (AddGene, 12260). 48h after transfection, culture supernatants containing virus were harvested and filtered. The filtrate was concentrated by ultra-centrifuging for 2h at 23,000 RPM, 4°C. Cells were infected with the concentrated viral supernatants and 8 mg/mL polybrene. For stable knockdown, 3 days after viral infection, cells were selected in 0.5 mg/mL puromycin. Puromycin-resistant cells were seeded in MethoCult®. Cell clones were picked up and were characterized by western blots to determine if BCL-2 was significantly reduced (knockdown) or completely depleted (knock out) after gRNA transduction and selection.

Cell Viability Assays

5000 cells were seeded in 96-well plates in 50µL RPMI medium in triplicate. 50µL drug medium at five or nine serial diluted concentrations were added to the cell suspension, with each concentration having three replicates per cell line. After a 72h incubation, 20µl of Resazurin reagent (R&D Systems, AR002) was added into the 96-well plates. The plates were read after a 2h incubation using 560/590 nm wavelength to estimate cell proliferation. Relative cell viability was normalized to DMSO treated wells. Experiments were repeated at least 3 times.

High-Throughput Small-Molecule Drug Screens

Using a semi-automated platform, we tested the potency of a 31 small molecule annotated library in paired parental and S63845-resistant derivatives of SUDHL-16, Mino, and HBL-2 cells. Cell viability was estimated by using Resazurin. In brief, cells were seeded in 384-well plates with 2000 cells per well in 90μl medium. Cells were cultured in the presence of different compounds at serial threefold diluted concentrations with each condition having duplicate wells on a single plate. After 3 days of treatment, 10μl of Resazurin reagent was added into each well and incubated at 37°C in 5% CO_2 for 2 hours. Plates were read at 560/590 nm wavelength to estimate cell proliferation. Relative cell viability was normalized to DMSO treated wells. Experiments were repeated at least 3 times.

Cell-Based Imaging Analysis of Drug Screening Assay

Cells were seeded in a 384-well plates of a reconstructed lymphoma TME, including high physiological densities (1-10 x 10^6 cells/ml), extracellular matrix (collagen, fibronectin), and lymphoma stromal cells (HK cells or autologous stromal cells). A panel of drugs at five serial diluted concentrations were added to the media with each condition having duplicate wells on a single plate, and plates were continuously imaged every 30 mins for 96 hours (cell line) or 144 hours (primary sample). All images were analyzed using a digital image analysis algorithm to detect cell viability based on membrane motion (pseudo-colored in green), and changes in viability were quantified by area under curve (AUC) and EC50 as described (2-4). Experiments were repeated at least 3 times.

RNA-Sequencing

All samples were prepared in biological triplicates. Total RNA was isolated using the RNeasy Plus Mini (Qiagen Cat# 74134). Library prep was conducted using TruSeq Stranded mRNA Library Prep Kit (Illumina Cat #RS-122-2101/2) according to the manufacturer's instructions. RNA sequencing was performed on HiSeq 2500v4 high output (50-bp, single-end reads). Tophat2 was used to align the Fastq files. TPM values were calculated and normalized using Cuffnorm. Genes that had a $p < 0.05$ and at least a 1.5-fold change were considered to be significantly altered between sensitive and resistant phenotypes. Cutoff value for expressed genes was a TPM value greater than or equal to 1. Raw data for the primary MCL samples has been deposited in Gene Expression Omnibus under accession number GSE141336. Raw data from the cell line experiment has been deposited in Gene Expression Omnibus under accession number GSE160742.

Activity-Based Protein Profiling (ABPP)

All samples were prepared in biological triplicates. Briefly, cell pellets were sonicated in IP/Lysis buffer, desalted and then depleted of endogenous ATP with Zeba spin column, and incubated with 10μM desthiobiotin-ATP probes at room temperature for 10 min. The labeled proteins were reduced, alkylated and trypsin digested at 37°C for 2 hr. The labeled peptides were purified with high capacity streptavidin agarose resin, washed, eluted and subjected to LC-MS/MS for peptide sequencing. The peptide identification and relative quantification were performed using MaxQuant software (Version 1.2.2.5). The procedures of ABPP were as detailed previously (2). Missing values were imputed using the Perseus software (Version 1.6.5.0). Peptides with a 1.5-fold change or higher were highlighted in the kinome tree, drawn through Kinmap.

Gene Set Enrichment Analysis (GSEA)

Gene set enrichment analysis (GSEA) was performed as described (5). The most differentially expressed genes ranked by $\log_2$ fold change for each comparison were used to generate a signature for GSEA analysis. GSEA estimates if genes are specific to either parental or resistant cells, indicating they are associated with a specific phenotype, rather than being distributed uniformly or randomly across the list. An enrichment score (ES) is calculated to quantify the degree to which a gene set is over-represented at the top or bottom of the entire ranked list. After calculation of the scores for a collection of gene sets, an empirical phenotype-based permutation test procedure is used to estimate P-values. GSEA normalizes the ES for each gene set to account for the variation in set sizes, yielding a normalized enrichment score (NES) and a false discovery rate (FDR). The FDR gives an estimate of the probability that a set with a given NES represents a false positive finding; it is computed by comparing the tails of the observed and permutation-computed null distributions for the NES.

Statistics

Unless otherwise stated, comparison and statistical significance between two groups in this paper are based on two-sided Student's t-test. P-values of less than 0.05 were considered significant. Data are shown with the mean ± SD of at least 3 experiments. Analysis of variance (ANOVA) or the Friedman Conover test was used for comparing data from multiple groups.

Code Availability

All algorithms and codes used to process images and perform downstream analyses for the image-based cell viability platform were provided directly by the Silva Lab and can be found online at http://www.i-genics.com/Jove2014Silva/ (4).

Supplementary Figure 1

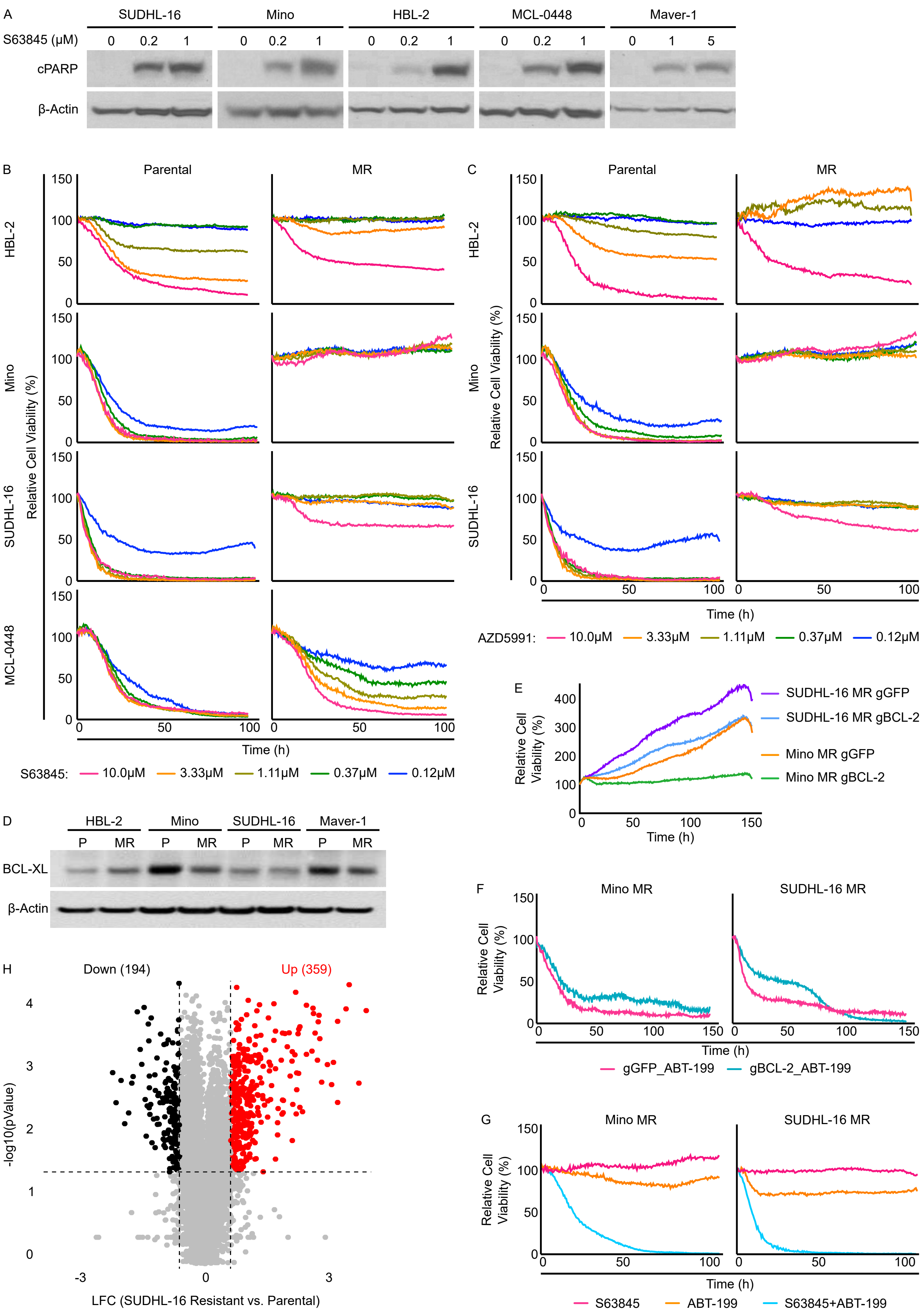

Supplementary Figure 1 (Related to Figure 1). MCL-1 dependency and development of resistance to MCL-1 inhibition in mantle cell lymphoma (MCL) and diffuse large B-cell lymphoma (DLBCL). **A.** Western blot analysis of S63845 treatment triggering PARP cleavage in S63845 sensitive cell lines in a dose-dependent fashion. cPARP: cleaved PARP. **B.** Image-based cell-viability assays of S63845 response in paired parental (left) and MR (right) cell lines. **C.** Image-based cell-viability assays of AZD5991 response in paired parental (left) and MR (right) cell lines. **D.** Western blot analysis of BCLXL protein expression in paired parental and MR cell lines. **E.** Image-based cell-viability assays of MR cells with and without BCL-2 knockdown in the absence of drug. **F.** Image-based cell-viability assays of MR cells with and without BCL-2 knockdown in response to ABT-199 (Left: 10μM, Right: 0.12μM). **G.** Image-based cell-viability assays of MR cells treated with S63845 (1.11μM), ABT-199 (0.12μM), or S63845 (1μM) + ABT-199 (0.12μM). **H.** Volcano plot of differentially expressed genes in SUDHL-16 MR cells compared to parental cells. Red: Upregulated genes, black: downregulated genes. LFC: $\log_2$ fold change cut-off of $\log_2(1.5)$, P-value cut-off of 0.05. n = 3 biologically independent samples. Data shown in **A** through **D** and **G** are representative of at least 3 independent experiments.

Supplementary Figure 2

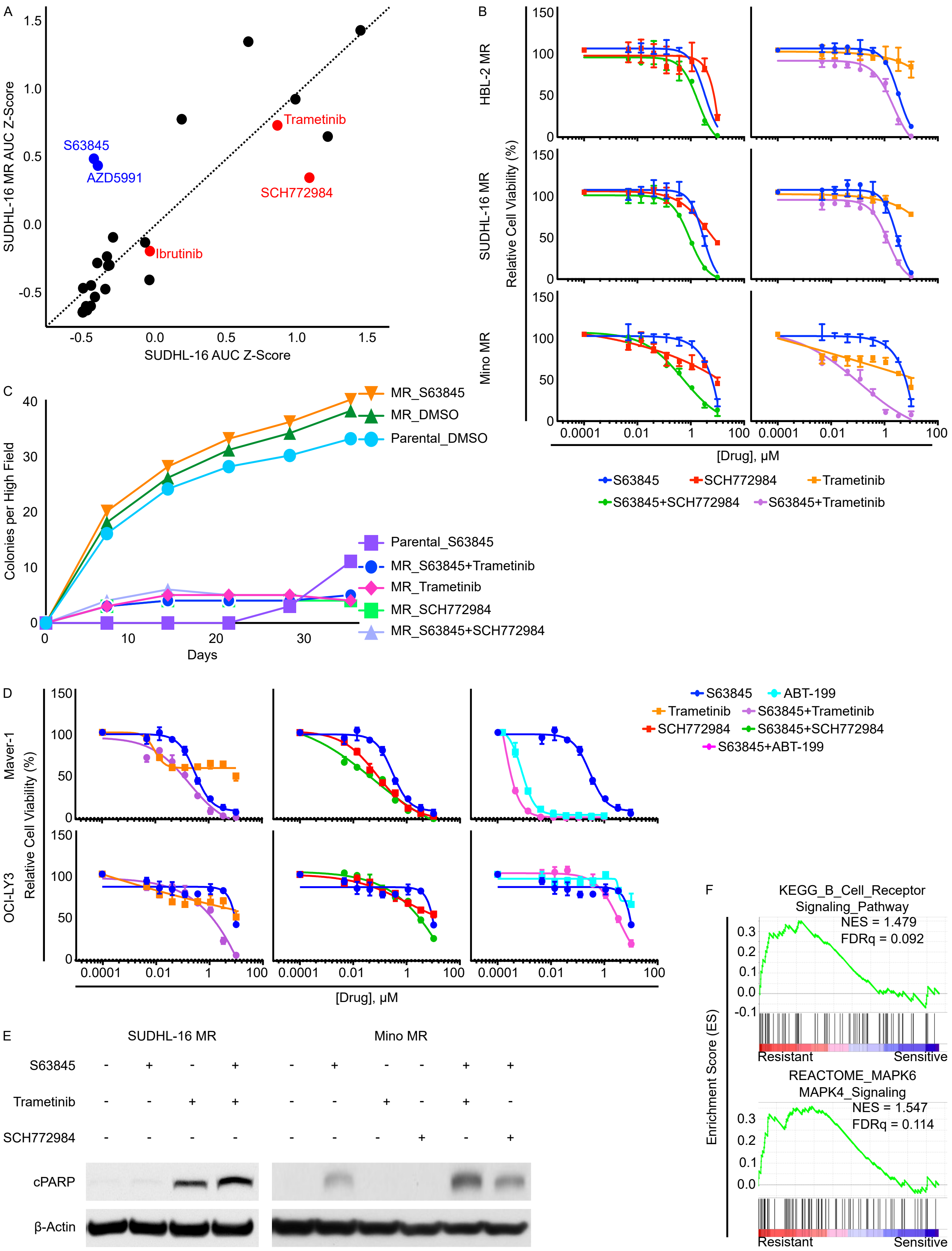

Supplementary Figure 2 (Related to Figure 2). MCL-1 as a novel vulnerability for combination therapy in mantle cell lymphoma (MCL) and diffuse large B-cell lymphoma (DLBCL). A. Z-scores of normalized AUCs of drug response curves from high-throughput semi-automated small-molecule drug screen performed in SUDHL-16 Parental and MR cells. Selected compounds with decreased potencies in MR cells are highlighted in blue and increased potencies in MR cells are highlighted in red. **B.** Left: Dose-response curves of indicated MR cell lines after 72h treatment with S63845, SCH772984, or S63845+SCH772984 at different doses. Right: Dose-response curves of indicated MR cell lines after 72h treatment with S63845, Trametinib, or S63845+Trametinib at different doses. Data is shown as mean ± SD, n = 3 technical replicates for each cell line. **C.** Clonogenic growth assay of parental and MR cells treated with DMSO, S63845 (500nM), SCH772984 (200nM), Trametinib (200nM), S63845+SCH772984 (500nM and 200nM, respectively), or S63845+Trametinib (500nM and 200nM, respectively), for the indicated time points. **D.** Dose-response curves of MCL cell line Maver-1 (top) and DLBCL cell line OCI-LY3 (bottom) treated with S63845, SCH772984, or S63845+SCH772984 (left), S63845, Trametinib, or S63845+Trametinib (middle), and S63845, ABT-199, or S63845+ABT-199 (right) at different doses. **E.** Western blot analysis of PARP cleavage in MR cell lines after 48 hours of indicated drug treatments at 1μM (left) and 2μM (right) of each drug. **F.** GSEA enrichment score plots of selected pathways from KEGG and Reactome (C2) gene sets from MSigDB. Primary patient samples were phenotypically categorized by S63845 sensitivity as determined by AUCs calculated from image-based cell-viability assays. Data shown in **A**, **B**, **D**, and **E** are representative of at least 3 independent experiments.

References

1. Kwiatkowski N, et al. Targeting transcription regulation in cancer with a covalent CDK7 inhibitor. Nature. 2014;511(7511):616-20.

2. Zhao X, et al. Unification of de novo and acquired ibrutinib resistance in mantle cell lymphoma. Nat Commun. 2017;8:14920.

3. Silva A, et al. An Ex Vivo Platform for the Prediction of Clinical Response in Multiple Myeloma. Cancer Res. 2017;77(12):3336-51.

4. Silva A, Jacobson T, Meads M, Distler A, Shain K. An Organotypic High Throughput System for Characterization of Drug Sensitivity of Primary Multiple Myeloma Cells. J Vis Exp. 2015(101):e53070.

5. Subramanian A, et al. Gene set enrichment analysis: a knowledge-based approach for interpreting genome-wide expression profiles. Proc Natl Acad Sci U S A. 2005;102(43):15545-50.
